# The Roles of IL-22 and Its Receptor in the Regulation of Inflammatory Responses in the Brain

**DOI:** 10.3390/ijms23020757

**Published:** 2022-01-11

**Authors:** Dahae Lee, Hyejung Jo, Cheolhyeon Go, Yoojin Jang, Naghyung Chu, Suhyun Bae, Dongmin Kang, Yejin Kim, Jae Seung Kang

**Affiliations:** 1 Laboratory of Vitamin C and Antioxidant Immunology, Department of Anatomy and Cell Biology, Seoul National University College of Medicine, Seoul 03080, Korea; ddhh12345@snu.ac.kr (D.L.); luv_jo@snu.ac.kr (H.J.); rhcjfgus@snu.ac.kr (C.G.); pierce52@snu.ac.kr (Y.J.); bsh9706@gmail.com (S.B.); 2 Department of Biology, College of Arts and Sciences, Emory University, Atlanta, GA 30322, USA; nchu0423@gmail.com; 3 Department of Psychological and Brain Sciences, College of Arts and Sciences, Boston University, Boston, MA 02215, USA; dong1109@bu.edu; 4 Medical Research Center, Institute of Allergy and Clinical Immunology, Seoul National University, Seoul 03080, Korea

**Keywords:** IL-22, IL-22Rα, inflammation, BV2, HT22

## Abstract

Interleukin (IL)-22 is a potent mediator of inflammatory responses. The IL-22 receptor consists of the IL-22Rα and IL-10Rβ subunits. Previous studies have shown that IL-22Rα expression is restricted to non-hematopoietic cells in the skin, pancreas, intestine, liver, lung, and kidney. Although IL-22 is involved in the development of inflammatory responses, there have been no reports of its role in brain inflammation. Here, we used RT-PCR, Western blotting, flow cytometry, immunohistochemical, and microarray analyses to examine the role of IL-22 and expression of IL-22Rα in the brain, using the microglial cell line, hippocampal neuronal cell line, and inflamed mouse brain tissue. Treatment of BV2 and HT22 cells with recombinant IL-22 increased the expression levels of the pro-inflammatory cytokines IL-6 and TNF-α, as well as cyclooxygenase (COX)-2 and prostaglandin E2. We also found that the JNK and STAT3 signaling pathways play an important role in IL-22-mediated increases in inflammatory mediators. Microarray analyses revealed upregulated expression of inflammation-related genes in IL-22-treated HT22 cells. Finally, we found that IL-22Rα is spontaneously expressed in the brain and is upregulated in inflamed mouse brain. Overall, our results demonstrate that interaction of IL-22 with IL-22Rα plays a role in the development of inflammatory responses in the brain.

## 1. Introduction

Interleukin (IL)-22, a member of the IL-10 cytokine family, is produced by several subsets of lymphocytes, including CD4 + T helper 17 (Th17) and Th22 cells, natural killer cells, CD8+ cytotoxic T cells, γδ T cells, and lymphoid tissue inducer-like cells [1,2,3,4,5]. In addition, it has been reported recently that IL-22 is also produced by activated macro-phages [6]. Although IL-22 has an anti-inflammatory role in inflammatory bowel diseases, it can promote inflammatory conditions in autoimmune diseases such as rheumatoid arthritis (RA), Crohn’s disease, and various skin diseases [7,8]. Also, IL-22 upregulates the production of acute-phase proteins in hepatoma cells, suggesting that it is involved in the regulation of inflammatory responses [4]. The biological role of IL-22 was originally described in hepatoma cells, keratinocytes, and pancreatic acinar cells, and it was subsequently reported to be involved in the pathogenesis of numerous inflammatory diseases, notably psoriasis [9,10,11].

The IL-22 receptor (IL-22R) is a heterodimer of IL-22Rα and IL-10Rβ subunits [5]. IL-10Rβ is expressed in a wide variety of cells, including immune cells, whereas IL-22Rα expression is thought to be limited to epithelial cells in organs such as the lung, kidney, colon, pancreas, and skin [4,12,13]. For this reason, the roles of IL-22Rα have typically been studied using non-hematopoietic organs [14]. These studies have shown that binding of IL-22 to IL-22Rα induces activation of Janus kinase 1 (JAK1), and the signal transducers and activators of transcription protein 3 (STAT3) and STAT5 pathways, as well as MAP kinase pathways such as the extracellular signal regulated kinase (ERK1/2), c-Jun N-terminal kinase (JNK), and p38 pathways [2,15,16,17].

To our knowledge, there have been no reports describing expression of IL-22Rα in the brain. Therefore, we examined both the role of IL-22 and expression of IL-22Rα in the brain using the BV2 microglial cell line, HT22 hippocampal neuronal cell line, and experimentally inflamed mouse brain tissue [18,19,20]. As the resident macrophages of the central nervous system (CNS), microglia play an important role in the development of inflammatory responses in the brain [19,21]. These specialized cells are generally considered to originate from bone marrow-derived monocytes, and are exquisitely sensitive to brain diseases and injury, altering their morphology and phenotype to adopt an activated state in response to pathophysiological brain insults [22,23,24]. Activated microglia display retracted processes and enlarged cell bodies, and become proliferative at the injured brain site [18,22,25]. The cytotoxic effects of microglia and their inflammatory roles mediated through the production of IL-6, IL-1β, and TNF-α implicate them in a variety of pathological states [26,27]. Activated microglia release various bioactive molecules, including nitric oxide and reactive oxygen species. In addition, cyclooxygenase-2 (COX-2) is an enzyme that produces prostaglandin E2 (PGE2), a major inducer of inflammatory processes in the hippocampus [28,29,30,31]. Hence, we examined the effects of IL-22 on IL-6, TNF-α, COX-2, and PGE2 expression in mouse brain cell lines. We also used microarray analyses to identify the effects of IL-22 on the expression levels of inflammatory genes in HT22 cells.

## 2. Results

### 2.1. IL-22Rα Is Constitutively Expressed in BV2 Murine Microglial Cells, HT22 Hippocampal Neuronal Cells, and Mouse Brain Tissue

**Figure 1:**
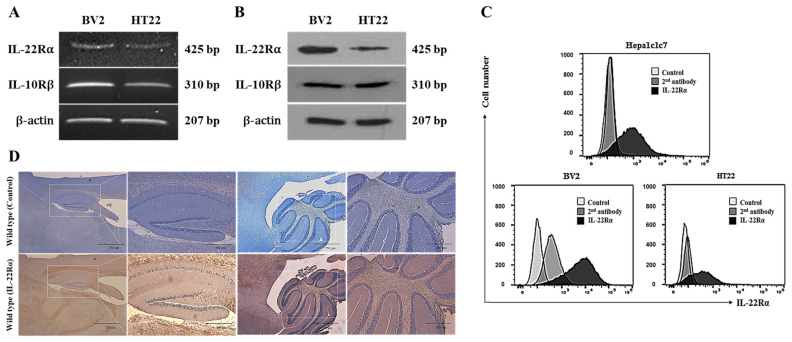
IL-22Rα is constitutively expressed in BV2 and HT22 cells and mouse brain tissue. (**A**) RT-PCR analyses of IL-22Rα and IL-10Rβ in BV2 and HT22 cells. Total RNA was extracted from cells (1 × 10^6^), and RT-PCR was performed using specific primers for IL-22Rα and IL-10Rβ, as described in the Materials and Methods. (**B**) Western blot analyses of IL-22Rα and IL-10Rβ in BV2 and HT22 cells. Protein was extracted from 1 × 10^6^ cells and analyzed with -IL-22Rα Ab and anti-IL-10Rβ Ab, as described in the Materials and Methods. β-actin was used as a loading control. (**C**) Flow cytometry analyses of IL-22Rα in BV2, HT22, and Hepa1c1c7 cells. For each sample, 1 × 10^5^ cells were collected, processed as described in the Materials and Methods, and then stained with an anti-mouse IL-22Rα antibody (2.5 µg/10^6^ cells) as a primary antibody, and FITC-conjugated anti-rabbit Ab was used as a secondary antibody. IL-22Rα expression was analyzed as described in the Materials and Methods. (**D**) Immunohistochemical analysis of IL-22Rα expression in mouse brain. Paraffin-embedded tissues were sectioned with 4 μm thickness and incubated with a primary antibody against IL-22Rα and then with a biotinylated anti-rabbit antibody. ABC solution was loaded onto the sections for 30 min and a DAB kit was used for chromogenic detection. (**A**–**D**) Results are representative of three independent experiments.

It is known that IL-22 shows its activity through the binding with IL-22R composed of IL-22Rα and IL-10Rβ. The expression of IL-22Rα is induced and increased under inflammatory condition. IL-10Rβ is constitutively expressed regardless with inflammation and plays a crucial role for signal transduction through IL-22R after dimerization with IL-22Rα. RT-PCR and Western blotting analyses revealed that IL-22Rα is expressed endogenously in BV2 murine microglial cells and HT22 hippocampal neuronal cells and IL-10Rβ is constitutively expressed (Figure 1A,B). A flow cytometry analysis also confirmed that IL-22Rα is expressed on the surface of BV2 and HT22 cells, as well as on that of Hepa1c1c7 murine hepatoma cells (used as a positive control; Figure 1C). In addition, an immunohistochemical analysis revealed that IL-22Rα is constitutively expressed in mouse brain tissues, especially the hippocampus and cerebellum, and its expression is increased by inflammation (Figure 1D). It is known that IL-22 shows its activity through the binding with IL-22R composed of IL-22Ra and IL-10Rb.

### 2.2. The Interaction of IL-22 with IL-22Rα Induces Proinflammatory Cytokine Production in BV2 and HT22 Cells

**Figure 2:**
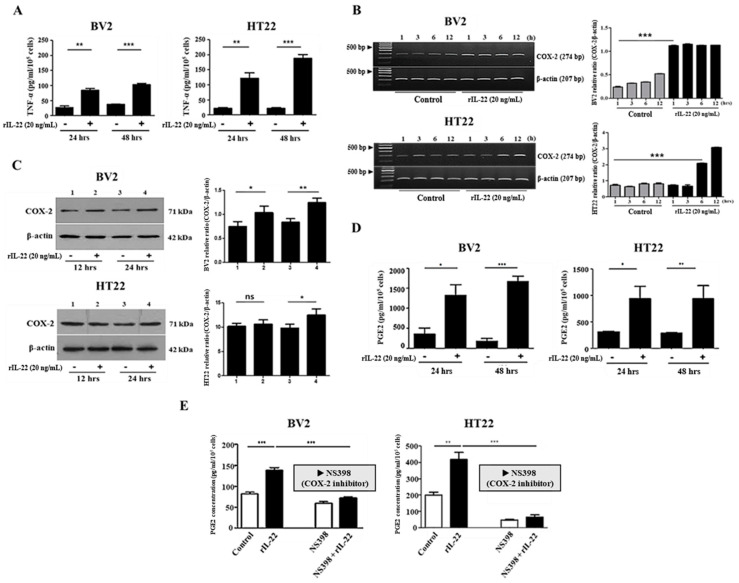
The interaction between IL-22 and IL-22Rα induces pro-inflammatory cytokine production in BV2 and HT22 cells. (**A**) ELISA-based analysis of TNF-α in the supernatants of BV2 and HT22 cells treated with or without IL-22 (20 ng/mL) for 24 or 48 h. ** *p* < 0.01; *** *p* < 0.001. (**B**) RT-PCR analysis of COX-2 expression in BV2 and HT22 cells treated with or without IL-22 (20 ng/mL) for 1, 3, 6, or 12 h. Relative intensity was analyzed by ImageJ software. All results were representative of at least three independent experiments. Values were presented as the mean ± SD. Significance (*p*-value) was determined by *t*-test, *** *p* < 0.001. (**C**) Western blot analysis of COX-2 expression in BV2 and HT22 cells treated with or without IL-22 (20 ng/mL) for 12 or 24 h. Relative intensity was analyzed by ImageJ software. * *p* < 0.05; ** *p* < 0.01; ns, not significant. (**D**) ELISA-based analysis of PGE2 in the supernatants of BV2 and HT22 cells treated with or without IL-22 (20 ng/mL) for 24 or 48 h. * *p* < 0.05; ** *p* < 0.01; *** *p* < 0.001. (**E**) ELISA-based analysis of PGE2 in the supernatants of BV2 and HT22 cells treated with or without IL-22 (20 ng/mL) and/or NS-398 (40 μM) for 24 h. ** *p* < 0.01; *** *p* < 0.001.

Since IL-22 is an important pro-inflammatory cytokine, we investigated whether it induces the production of IL-6 and TNF-α in BV2 and HT22 cells via interaction with IL-22Rα [14]. As shown in Figure 2A, IL-22 treatment increased TNF-α production significantly in both cell lines. COX-2 is rarely expressed in steady state, but it is rapidly upregulated under inflammatory conditions. For this reason, we examined the effects of IL-22 treatment (20 ng/mL) for 1, 3, 6, or 12 h on COX-2 mRNA expression in BV2 and HT22 cells. RT-PCR analyses revealed that COX-2 mRNA expression was increased remarkably in both cell lines at 1 and 6 h after IL-22 treatment (Figure 2B). In addition, Western blotting analyses revealed that COX-2 protein levels were increased significantly in both cell lines 24 h after IL-22 treatment (Figure 2C). Next, the effect of exposure to IL-22 (20 ng/mL) for 12 or 24 h on PGE2 production was examined by ELISA. As expected, PGE2 production by BV2 and HT22 cells was increased significantly at 24 and 48 h after the treatment (Figure 2D). To examine whether IL-22 increases PGE2 production via the activation of COX-2, PGE2 production was measured after the treatment of NS-398, a specific inhibitor of COX-2, on both cells. As a result, the IL-22-induced increase in PGE2 production was attenuated by the treatment of NS-398 (Figure 2E).

### 2.3. The JNK and STAT3 Signaling Pathways Play an Important Role in IL-22-Induced Proinflammatory Cytokine Production in BV2 and HT22 Cells, Respectively

**Figure 3:**
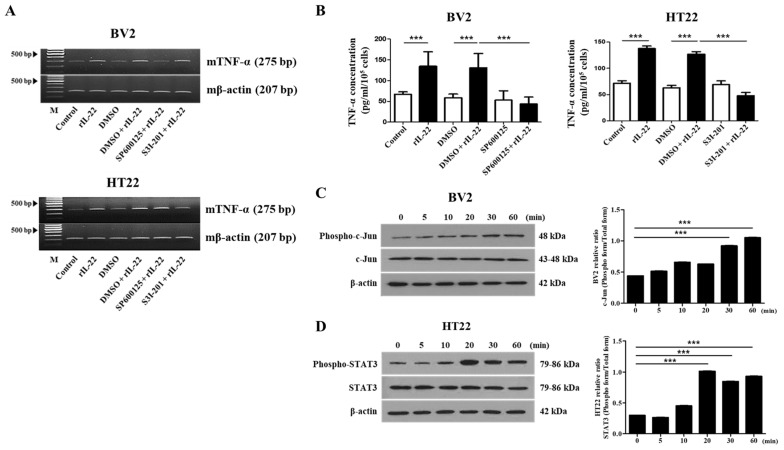
The JNK and STAT3 signaling pathways play an important role in IL-22-mediated inflammatory cytokine production by BV2 and HT22 cells, respectively. (**A**) RT-PCR analyses of TNF-α in cells that were pretreated with DMSO (vehicle control), SP600125 (20 μM), and S3I-201 (50 μM) for 1 h prior to treatment with IL-22 (20 ng/mL) for 12 h. (**B**) ELISA-based analysis of TNF-α in the supernatants of cells that were pretreated with DMSO (vehicle control), SP600125 (20 μM), or and S3I-201 (50 μM) for 1 h prior to treatment with IL-22 (20 ng/mL) for 48 h. *** *p* < 0.001. (**C**) Immunoblot analyses of c-Jun and phosphorylated c-Jun in BV2 cells that were pretreated with SP600125 (20 μM) and then treated with IL-22 (20 ng/mL) for 0, 5, 10, 20, 30, or 60 min. Relative intensity was analyzed by ImageJ software. All results were representative of at least three independent experiments. Values were presented as the mean ± SD. Significance (*p*-value) was determined by *t*-test, *** *p* < 0.001. (**D**) Immunoblot analyses of STAT3 and phosphorylated STAT3 in HT22 cells that were pretreated with S3I-201 (50 μM) and then treated with IL-22 (20 ng/mL) for 0, 5, 10, 20, 30, or 60 min. (**A**,**C**,**D**) Results are representative of three independent experiments. Relative intensity was analyzed by ImageJ software. All results were representative of at least three independent experiments. Values were presented as the mean ± SD. Significance (*p*-value) was determined by *t*-test, *** *p* < 0.001.

In view of the fact that IL-22 induces the production of pro-inflammatory cytokines, we sought to determine which signaling pathways are involved in this response. To this end, BV2 and HT22 cells were pretreated with SP600125 (20 μM), a specific inhibitor of JNK, and with S3I-201 (50 μM), a specific inhibitor of STAT3, prior to treatment with IL-22. RT-PCR analyses revealed that SP600125 inhibited IL-22-induced TNF-α expression in BV2 cells, and that S3I-201 inhibited TNF-α expression in HT22 cells (Figure 3A). These findings were also confirmed by ELISA (Figure 3B). In addition, IL-22 treatment (20 ng/mL) increased the phosphorylation of JNK in BV2 cells (Figure 3C) and the phosphorylation of STAT3 (Figure 3D) in HT22 cells.

### 2.4. IL-22Rα Expression Is Increased in the Gulo (-/-) Mouse Brain upon Inflammation

**Figure 4:**
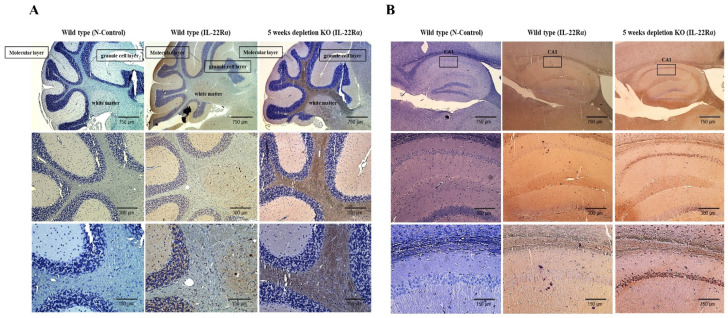
IL-22Rα expression is increased in the Gulo (-/-) mouse brain upon inflammation. (**A**) Cerebellum. (**B**) Hippocampus, CA1 region is localized to the stratum pyramidal and apical dendritic arborization extending into the stratum radiatum. Immunohistochemical staining of the sagittal sections of the WT and 5 weeks Gulo (-/-) mouse brain and then paraffin-embedded tissues were sectioned with 4 μm thickness and incubated with a primary antibody against IL-22Rα and a biotinylated anti-rabbit antibody. Nuclei were counterstained with hematoxylin. Scale bar, 150 µm.

In our previous study [32], we used Gulo (-/-) mice lacking vitamin C supplementation for 5 weeks as a model for spontaneous brain inflammation, and found that vitamin C deficiency is related to defects in both motor and memory functions and the pathogenesis of neurodegenerative disorders, such as Huntington’s and Parkinson’s diseases. Therefore, we used vitamin C-deficient mice to examine the effects of inflammation on IL-22Rα expression in the brain. As shown in Figure 4A,B, IL-22Rα expression was increased in the cerebellum white matter region and the hippocampus CA1 region during the inflammatory response induced by vitamin C deficiency. These results suggest that inflammation induces IL-22Rα production in the cerebellum and hippocampus of Gulo (-/-) mice.

### 2.5. Inflammatory Genes Are Upregulated by IL-22 Treatment of HT22 Cells

**Figure 5:**
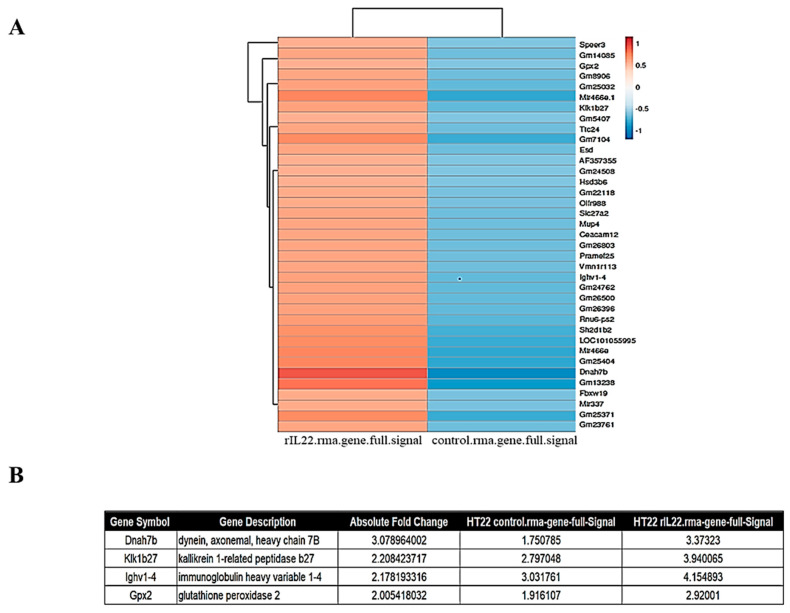
Gene expression profiling of control versus IL-22-treated HT22 cells. (**A**) Heatmap displaying individual differentially expressed genes (*n* = 109) by microarray analysis using Affymetrix GeneChip^®^ Mouse Gene 2.0 ST Arrays. (**B**) Subset of the inflammatory-related genes (Dnah7b, Klk1b27, Ighv1-4 and Gpx2) with large log2 fold-changes between IL-22-treated and control samples (*n* = 4).

Next, we used Affymetrix GeneChip^®^ Mouse Gene 2.0 ST Arrays to examine the expression levels of inflammatory genes in HT22 cells following IL-22 treatment. Of the 109 genes analyzed (Figure 5A), 37 genes were upregulated following IL-22 treatment, and four of these genes were identified as inflammatory-related factors (Figure 5B). These genes included dynein, axonemal, heavy chain 7B (Dnah7b) [33], kallikrein 1-related peptidase b27 (Klk1b27) [34], immunoglobulin heavy variable 1-4 (Ighv1-4) [35,36], and glutathione peroxidase 2 (Gpx2) [37]. This section may be divided by subheadings. It should provide a concise and precise description of the experimental results, their interpretation, as well as the experimental conclusions that can be drawn.

## 3. Discussion

As a cytokine belonging to the IL-10 family, IL-22 is released from activated T cells and natural killer cells [9,11]. In addition, it has been reported recently that IL-22 is produced by activated macrophages [38,39,40]. Although IL-22 can have both pro-inflammatory and anti-inflammatory roles, its pro-inflammatory roles have been studied most extensively, and several studies have shown correlations between IL-22 production and inflammatory skin conditions, uveitis, Alzheimer’s disease, and liver injury [7,11,12]. Nonetheless, IL-22 has an anti-inflammatory effect on inflammatory bowel diseases [41,42]. The cell surface-standing IL-22 receptor complex consists of the IL-22Rα and IL-10Rβ receptor chains [5]. IL-10Rβ is expressed in all cells, including immune cells, whereas IL-22Rα is reportedly expressed in non-hematopoietic cells of the skin, kidney, intestine, liver, and pancreas [5,10,43]. To our knowledge, there have been no investigations of IL-22Rα expression in the brain; therefore, our current study examined IL-22 function and IL-22Rα expression in the mouse brain.

We examined IL-22Rα expression in the HT22 hippocampal neuronal cell line and the BV2 microglial cell line. The latter was selected because microglia protect the CNS by acting as the initial responders to infection [22,44,45]. Additionally, since IL-22Rα is released from the liver, we used the Hepa1c1c7 mouse hepatoma cell line as a positive control. We found that IL-22Rα was expressed at both the mRNA and protein level in BV2 and HT22 cells, and the levels of IL-22Rα in both neuronal cell lines were similar to that in Hepa1c17 cells (Figure 1A–C). IL-10Rβ expression was also confirmed in both cell lines. Previous studies have examined IL-22Rα expression at the cellular level and in non-hematopoietic tissues such as the skin and pancreas [9,11]. In addition to its expression in mouse brain cell lines, we also identified IL-22Rα expression in the hippocampus and cerebellum of C57BL/6 mice (Figure 1D). The hippocampus plays an important role in spatial memory and cognition, enabling information integration and transition from short-term memory to long-term memory [25,35,46]. In addition, the cerebellum is involved in motor control [32,47,48]. Therefore, defects of the hippocampus are related to Alzheimer’s disease and those of the cerebellum are related to Parkinson’s disease, both of which are inflammatory brain diseases [25,49]. With this in mind, we examined the effect of vitamin C deficiency-induced brain inflammation on IL-22Rα expression in mice. The brain and cerebrospinal fluid have high concentrations of vitamin C, and oxidative stress in relation to the therapeutic function of vitamin C has been implicated to develop neurodegenerative diseases [32]. In our current study, a 5-week deficiency of vitamin C created an inflammatory environment in mice and upregulated expression of IL-22Rα in the cerebellum white matter region, as well as the CA1 region of the hippocampus. Overall, these findings demonstrate that IL-22Rα is expressed in the brain and is upregulated during inflammatory responses.

It is known that IL-22 shares its function with IL-17 in terms of epithelial protection and regeneration of skin, lung, and gastrointestinal tract [9,14]. It was recently reported by Zhao, N. et al. that the modulation of Th17/Treg imbalance in CSE-induced experimental COPD is achieved by the inhibition of IL-22-dependent JAK/STAT3 pathway [50]. While IL-17 shows its function as a pro-inflammatory cytokine, IL-22 has roles of both an anti-inflammatory cytokine as well as pro-inflammatory cytokine. Even though IL-22 plays as an anti-inflammatory cytokine in IBD and sepsis-induced liver injury [3], its role in the inflamed-brain is still not elucidated. That is why we examined the inflammatory role of IL-22 on brain inflammation in this study. In case of inflammation in brain, it seems that Th17/Treg imbalance is not directly involved in the development and pathogenesis of inflammation in brain, since T cell is not resided in the brain. However, it is worthy to examine the expression of IL-17R, like the expression of IL-22Rα, on neuronal cells, especially cells in animal models with Parkinson’s disease and Alzheimer’s disease.

As IL-22 is involved in the induction of inflammatory responses in other tissues, we investigated its effects on the production of inflammatory cytokines [41,51], and found that treatment of BV2 and HT22 cells with IL-22 increased the levels of TNF-α (Figure 2A) and IL-6 (Figure S1). Previous studies have shown that binding of IL-22 to IL-22Rα induces the JAK1, STAT3, and STAT5 pathways, as well as the MAP kinase pathways [17,52,53]. In line with these reports, we found that pretreatment of BV2 cells with SP600125 (a specific inhibitor of JNK) and pretreatment of HT22 cells with S3I-201 (a specific inhibitor of STAT3) attenuated the IL-22-induced increase in TNF-α (Figure 3A,B). At the same time, the phosphorylation of JNK (Figure 3C) and phosphorylation of STAT3 (Figure 3D) were increased in IL-22-treated BV2 and HT22 cells, respectively. Overall, these findings suggest that IL-22 induces pro-inflammatory cytokine production in mouse brain cells and that the JNK pathway is involved in IL-22-induced TNF-α expression in BV2 cells, whereas the STAT3 pathway is involved in this response in HT22 cells. Even though the signaling pathways for TNF-α production in both cell lines are different, our results suggest that IL-22 might induce inflammation in the brain through TNF-α production. Considering the pathogenic role of STAT3 in the several kinds of inflammatory diseases, it also implies that the substances, which can suppress STAT3 activation, could be used more effectively for the regulation of IL-22-induced inflammation in the brain.

Inflammatory prostaglandin signaling plays a role in the preclinical development of neurodegenerative diseases [29,30,54]. COX-2, the inducible form of the COX enzyme involved in prostaglandin synthesis, is rarely expressed in steady state but is induced rapidly following stimulation by a mitogen, cytokine, or lipopolysaccharide [28,31,55]. Here, we found that COX-2 expression was increased following treatment of BV2 and HT22 cells with IL-22, and there was a time difference between the responses of the BV2 and HT22 cell lines at the transcriptional (Figure 2B) and translational levels (Figure 2C). In line with the fact that overexpression of COX-2 eventually leads to increased prostaglandin formation, we also found that PGE2 production was increased by treatment of BV2 and HT22 cells with IL-22 (Figure 2D). In addition, IL-22-induced PGE2 production was decreased in cells treated with NS-398, a COX-2 specific inhibitor (Figure 2E). Although microglia function to protect the body, over-activation can result in the production of IL-6, IL-1β, TNF-α, and other inflammatory cytokines, resulting in neuronal death and chronic inflammation, the major causes of degenerative brain disease [56,57].

Microarray gene expression profiling enables investigation of genome-wide changes in gene expression and provides an unbiased approach for identifying genes that are regulated under pathological conditions. Therefore, we used microarrays to identify genes that are differentially expressed in HT22 cells following treatment with IL-22. We found that the expression levels of the inflammatory genes Dnah7b, Klkb27, Ighv1-4, and Gpx2 were upregulated following IL-22 treatment (Figure 5). Dnah7b promotes androgen receptor activity and is expressed at high levels in primary tumors [58]. Kallikrein 1-related peptidase b27 (Klk1b27) is important for neuronal development and plasticity [59]. Immunoglobulin-heavy variable 1-4 (Ighv1-4) accounts for 85% of follicular lymphoma cases [60]. Glutathione peroxidase 2 (Gpx2) catalyzes the reduction of H2O2 by glutathione [34,61].

Inflammation in the brain contributes to the development of Alzheimer’s and Parkinson’s diseases, and pathological studies have shown that increased levels of COX-2, PGE2, IL-6, and TNF-α play a role in these degenerative brain diseases [62,63,64]. Notably, the levels of these factors were increased in IL-22-treated mouse neuronal cells. It is highly likely that these effects rely on the interaction of IL-22 with IL-22Rα. For this reason, experiments using animal models to examine the roles of IL-22 and IL-22Rα in the development and progression of Alzheimer’s and Parkinson’s diseases are warranted. We also found that treatment of BV2 and HT22 cells with IL-23 increased expression of IL-22Rα (data not shown: Material not intended for publication). Thus, regulation of IL-22Rα expression through the regulation of IL-23 may be a starting point for controlling inflammation in the brain and the consequent development of degenerative brain diseases such as Alzheimer’s and Parkinson’s. If IL-22Rα is already expressed at high levels, it may be possible to block its function using a neutralizing Ab or soluble IL-22 binding protein. Based on our unpublished data regarding the expression of IL-22 binding protein (BP), it was expressed only in BV2, but not in HT22 (Figure S2). Considering several reports that microglia have a role in the protection of neurons, it seems that IL-22BP has a protective role in IL-22-induced damage of neuron. Therefore, the additional experiment using animal models with inflammation in the brain should be needed.

This study was performed using cell lines and an animal model; follow-up studies using human brain tissue donated to the brain tissue bank are required to confirm our findings. Macrophages present in the brain and non-neuronal cells that are differentiated from monocytes and scattered in the CNS play an important role in brain inflammation. These large-sized cells cannot pass the blood–brain barrier and are difficult to examine using nanotechnology. Binding of IL-22 to IL-22Rα on the surface of BV2 and HT22 cells might play an important role in the proliferation of these cells and the production of inflammatory mediators.

## 4. Materials and Methods

### 4.1. Cell Lines and Culture Conditions

The BV2 murine microglial cell line, HT22 murine hippocampal neuronal cell line, and Hepa1c1c7 murine hepatoma cell line were maintained in DMEM (HyClone, Queensland, Australia) supplemented with 10% heat-inactivated fetal bovine serum (HyClone, Queensland, Australia) and antibiotics (100 U/mL penicillin and 100 μg/mL streptomycin; Welgene, Namcheon-myeon, South Korea) at 37 °C in a humidified atmosphere containing 5% CO2. The HT22 cell line was kindly provided by professor Inhee Mook-Jung (Seoul National University College of Medicine).

### 4.2. Animals

As described in our previous study [32], Gulo (-/-) mice (C57BL/6 background) lacking vitamin C supplementation for 5 weeks were used as a model of spontaneous brain inflammation. Gulo (-/-) mice were maintained in specific pathogen-free conditions in the animal facility at Seoul National University College of Medicine. The animal protocol was reviewed and approved by the Ethics Committee of Seoul National University (IACUC: SNU-200319-2).

### 4.3. Immunohistochemistry

Gulo (-/-) mice without vitamin C supplementation for 5 weeks were sacrificed and perfused with 4% paraformaldehyde (PFA). The brains were removed and post-fixed in 4% PFA at 4 °C overnight. Subsequently, the brains were transferred to 15% sucrose, placed at room temperature for 6 h, and then stored in 30% sucrose at 4 °C overnight. Fixed tissues were embedded in paraffin and sectioned with 4 µm thickness. After de-paraffinization and hydration, the antigen epitope was retrieved by heating with 0.1 M citrate buffer (pH 6.0) in a microwave. Subsequently, endogenous peroxidase was blocked with H2O2, and non-specific signals were blocked by incubating the sections with blocking solution (Vector Laboratories, Burlingame, CA, USA) containing 5% goat serum for 1 h at room temperature. To examine the expression level of IL-22Rα, tissue sections were incubated with a specific primary antibody (1:150; Abcam, Cambridge, UK) in a humidified chamber at 4 °C overnight. Subsequently, the sections were incubated with biotinylated goat anti-rat immunoglobulin as a secondary (1:250; Vector Laboratories) for 1 h at room temperature. ABC solution (Vector Laboratories) was loaded onto the sections for 30 min and a DAB kit (Vector Laboratories) was used for chromogenic detection. After counterstaining with hematoxylin, dehydration and clearing, tissue sections were mounted onto glass slides (Life Technologies, Frederick, MD, USA). An Olympus AX-70 microscope equipped with a motorized stage (Olympus, Melville, NY, USA) was used for visualization and data were analyzed using MCID 6.0 Elite Imaging Software (GE Healthcare, Piscataway, NJ, USA).

### 4.4. Flow Cytometry

BV2 and HT22 cells were resuspended in fluorescence-activated cell sorting (FACS) buffer containing 0.5% bovine serum albumin and blocked at 4 °C for 10 min with FcR blocking reagent (Miltenyi Biotec GmbH, Bergisch Gladbach, Germany). To examine the expression level of IL-22Rα, cells were incubated with a rabbit-derived polyclonal anti-IL-22Rα Ab (2.5 µg/10^6^ cells; Abcam) at 4 °C for 30 min. After washing the cells three times with FACS buffer and centrifuging at 1500 rpm (3 min each), the cells were stained with FITC-conjugated mouse-derived anti-rabbit IgG (1:400; Santa Cruz Bio-technology, Santa Cruz, CA, USA) on ice for 30 min. Subsequently, the cells were washed twice with FACS buffer (3 min each) and analyzed via Attune NxT Flow Cytometry (Thermo Scientific, Wilmington, DE, USA). FlowJo software (Tree Star, Ash-land, OR, USA) was used for data analysis.

### 4.5. Reverse Transcription-Polymerase Chain Reaction (RT-PCR)

Cells were pretreated with a specific inhibitor of JNK (SP600125, 20 μM; Sigma, St. Louis, MO, USA) or STAT3 (S3I-201, 50 μM; Sigma) for 1 h. After washing with PBS, the cells were treated with IL-22 (20 ng/mL) and cultured for a further 48 h. Total cellular RNA was extracted from BV2 and HT22 cells (1 × 10^6^) using TRIzol reagent (Invitrogen, Carlsbad, CA, USA). Reverse transcription was performed using 1 µg total RNA in a first-strand complementary DNA synthesis reaction with AMV Reverse Transcriptase (Promega, Madison, WI, USA). The sequences of the primers used for RT-PCR were as follows: 5′-CTG CAA CCT GAC TAT GGA GA-3′ (forward) and 5′-TTC ACT CGG CAC ACG TAG GG-3′ (reverse) for IL-22Rα (425 bp); 5′-AAG CAG AGT CCT GAA GAC AA-3′ (forward) and 5′-AGA TCA CTG TGA TCC TG-3′ (reverse) for IL-10Rβ (310 bp); 5′-ACA CAC TCT ATC ACT GGC ACC-3′ (forward) and 5′-TTC AGG GAG AAG CGT TTG C -3′ (reverse) for COX-2 (274 bp); 5′-TAC AGG CTT GTC ACT CGA ATT-3′ (forward) and 5′-ATG AGC ACA GAA AGC ATG ATC-3′ (reverse) for TNF-α (263 bp); and 5′-GAG AGT GGT GCC AGT CTA GT-3′ (forward) and 5′-GCC ACA CTC CAC AAT CA-3′ (reverse) for β-actin (207 bp). The PCR amplification conditions were as follows: 35 cycles of 94 °C for 15 s, 57 °C for 45 s, and 72 °C for 1 min (IL-22Rα); 30 cycles of 94 °C for 15 s, 58 °C for 45 s, and 72 °C for 1 min (IL-10Rβ); 35 cycles of 94 °C for 15 s, 55 °C for 45 s, and 72 °C for 1 min (COX-2); 40 cycles of 94 °C for 15 s, 57 °C for 45 s, and 72 °C for 1 min (TNF-α); and 35 cycles of 94 °C for 15 s, 58.1 °C for 45 s, and 72 °C for 1 min (β-actin). PCR products were separated by electrophoresis on a 1.5% agarose gel and visualized by staining with Red Safe (Intron Biotechnology, Seong-nam, Korea). The density of each band was analyzed using ImageJ software (NIH, Bethesda, MD, USA).

### 4.6. Western Blotting

BV2 and HT22 cells (1 × 10^6^) were lysed, and proteins were extracted using lysis buffer containing 50 mM Tris-HCL (pH 7.4), 1% NP-40, 0.25% sodium deoxycholate, 150 mM NaCl, 1 mM EDTA, and protease inhibitor cocktails. The protein concentration was measured using a BCA assay. Equal amounts of protein (30 μg/sample) were dissolved in a 10% polyacrylamide-SDS gel at 100 V for 2 h and transferred onto a nitrocellulose membrane. Blocking was performed at room temperature for 1 h with 5% non-fat milk in PBS containing 0.1% Tween 20 (PBST). To determine the expression levels of IL-22Rα and IL-10Rβ, the blocked membrane was incubated with an anti-IL-22Rα Ab (1:5000; Abcam), anti-IL-10Rβ Ab (1:5000; Santa Cruz Biotechnology), anti-phospho-c-Jun Ab (1:1000; Cell Signaling Technology, Boston, MA, USA), anti-c-Jun Ab (1:1000; Cell Signaling Technology), anti-phospho-STAT3 Ab (1:1000; Cell Signaling Technology), anti-STAT3 Ab (1:1000; Cell Signaling Technology), or anti-β-actin Ab (1:5000; Santa Cruz Biotechnology) at 4 °C overnight. After washing three times (5 min each) with 0.1% PBST, the membrane was incubated for 1 h at room temperature with horseradish peroxidase (HRP)-conjugated anti-rabbit IgG (1:10,000; Cell Signaling Technology) to detect IL-22Rα, c-Jun, or phospho-c-Jun, or with HRP-conjugated anti-mouse IgG (1:10,000; Cell Signaling) to detect IL-10Rβ, β-actin, phopsho-STAT3, and STAT3. Subsequently, the membrane was washed three times (5 min each), and the immunoreactive proteins were visualized with an electrochemical luminescence detection system (Thermo Scientific). The densities of the bands were analyzed using ImageJ software (NIH) and normalized to that of β-actin.

### 4.7. Enzyme-Linked Immunosorbent Assay (ELISA)

BV2 cells (1 × 10^5^) or HT22 cells (2 × 10^5^) were seeded into 6-well plates with or without recombinant (r)IL-22 (R&D Systems, Minneapolis, MN, USA) and/or NS-398 (40 μM; Sigma), and were allowed to grow to confluence for 24 or 48 h. Subsequently, the concentrations of IL-6, TNF-α (BioLegend, San Diego, CA, USA), and PGE2 (R&D Systems) in the supernatants were measured by ELISA, according to the manufacturers’ instructions. Relative absorbance was measured at 450 nm using a SpectraMax iD3 microplate reader (Molecular Devices, San Jose, CA, USA).

### 4.8. Gene Expression Profiling

Changes in gene expression profiles in HT22 cells treated with rIL-22 (20 ng/mL) were determined by microarray analysis using Affymetrix GeneChip^®^ Mouse Gene 2.0 ST Arrays. Total cellular RNA was extracted from 1 × 10^6^ cells using TRIzol reagent (Invitrogen). RNA quality was assessed using an Agilent 2100 bioanalyzer and the RNA 6000 Nano Chip (Agilent Technologies, Santa Clara, CA, USA), and quantity was determined using a Nanodrop-1000 spectrophotometer (Thermo Scientific). For microarray analyses, 300 ng RNA was used as input, as recommended by the Affymetrix protocol. The expression intensity data were extracted from the scanned images using Affymetrix Command Console software (version 1.1). After confirming that the data were normalized properly, genes that showed more than a 2-fold difference between the average signal values of the control and treatment groups were selected.

### 4.9. Statistical Analysis

Data are presented as the mean ± SD. Unpaired *t*-tests were used to compare two groups. Statistical analysis was carried out using GraphPad Software Prism version 6.01 (GraphPad Software, Le Jolla, CA, USA).

## 5. Conclusions

Our findings indicate that IL-22Rα is spontaneously expressed in brain cells, especially microglia and hippocampal neurons, and is involved in the development of inflammatory responses following binding of its ligand IL-22.

## Data Availability

All datasets generated for this study are included in the article or Supplementary File.

## Supplementary Materials

The following are available online at https://www.mdpi.com/article/10.3390/ijms23020757/s1.
