# Supplementary material for: The Roles of IL-22 and Its Receptor in the Regulation of Inflammatory Responses in the Brain
Source: Int J Mol Sci. 2022 Jan 11;23(2):757. doi: 10.3390/ijms23020757 (PMC8775345; doi:10.3390/ijms23020757)
Supplement: Supplementary file 1 [file ijms-23-00757-s001.zip › ijms-1521378-supplementary.pdf]

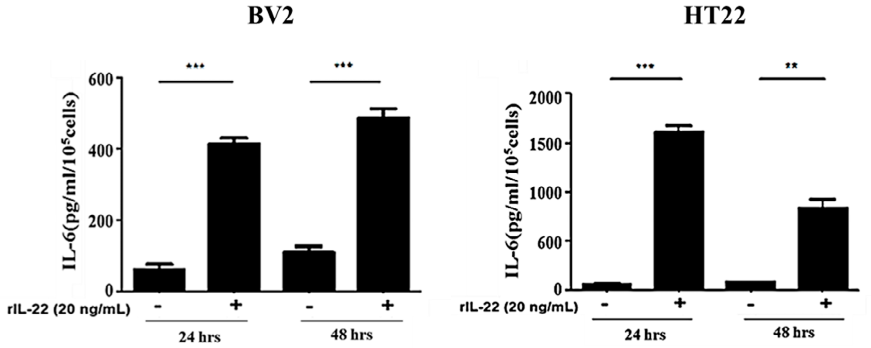

Figure S1. The interaction between IL-22 and IL-22Rα induces pro-inflammatory cytokine, IL-6 production in BV2 and HT22 cells. ELISA-based analysis of IL-6 in the supernatants of BV2 and HT22 cells treated with or without IL-22 (20 ng/mL) for 24 or 48 h. ** $P < 0.01$; *** $P < 0.001$.

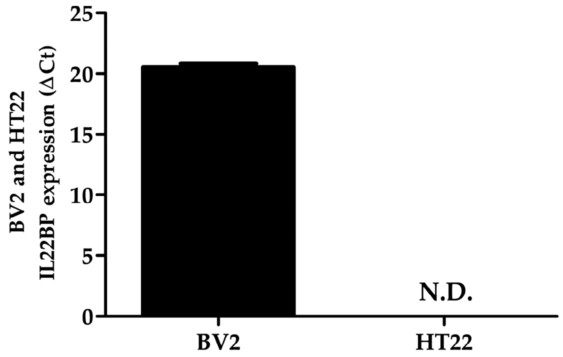

.

Figure S2. Total RNAs of BV2 and HT22 cells were isolated using Trizol Reagent (Invitrogen, CA, USA) and were reverse-transcribed into cDNA using oligo (dT) primers and AMV reverse transcriptase (INTRON, Daejeon, Korea). The real-time RT-PCR was processed with SYBR Green PCR master mix (MBI Fermentas, St. Leon-Rot, Germany) and performed using an ExiCyclerTM (Bioneer, Daejeon, Korea). The specific primers for mouse IL-22BP (5′-CATTGCCTTCTAGGTCTCCTCA-3′ and 5′-CCTGCTTGCCAGTGCAAAAT-3′) and mouse GAPDH (5′-TTGAGTCATCTCCAGCATCC-3′ and 5′-TTGAGTCATCTCCAGCATCC-3′). The expression of IL-22BP in BV2 and HT22 was calculated using the ΔCt method.
